# Secondary (additional) findings from the 100,000 Genomes Project: disease manifestation, healthcare outcomes and costs of disclosure

**DOI:** 10.1016/j.gim.2023.101051

**Published:** 2023-12-19

**Authors:** Joshua Nolan, James Buchanan, John Taylor, Joao Almeida, Tina Bedenham, Edward Blair, Suzanne Broadgate, Samantha Butler, Angela Cazeaux, Judith Craft, Treena Cranston, Gillian Crawford, Jamie Forrest, Jessica Gabriel, Elaine George, Donna Gillen, Ash Haeger, Jillian Hastings Ward, Lara Hawkes, Claire Hodgkiss, Jonathan Hoffman, Alan Jones, Fredrik Karpe, Dalia Kasperaviciute, Erika Kovacs, Sarah Leigh, Elizabeth Limb, Anjali Lloyd-Jani, Javier Lopez, Anneke Lucassen, Carlos McFarlane, Anthony W. O’Rourke, Emily Pond, Catherine Sherman, Helen Stewart, Ellen Thomas, Simon Thomas, Tessy Thomas, Kate Thomson, Hannah Wakelin, Susan Walker, Melanie Watson, Eleanor Williams, Elizabeth Ormondroyd

**Affiliations:** 1 Radcliffe Department of Medicine, University of Oxford, UK; 2 Health Economics Research Centre, University of Oxford, UK; 3 Oxford Genetic Laboratories, Oxford University Hospitals NHS Foundation Trust, Oxford, UK; 4 Genomics England, UK; 5 Oxford Centre for Genomic Medicine, Oxford University Hospitals NHS Foundation Trust, Oxford, UK; 6 Birmingham Women’s and Children’s Hospitals NHS Foundation Trust, Birmingham, UK; 7 University Hospitals Southampton NHS Foundation Trust, Southampton, UK; 8 University of Manchester, UK; 9 University Hospitals Birmingham NHS Foundation Trust, Birmingham, UK; 10 Participant Panel, Genomics England, UK; 11 Population Health Research Institute, St George’s University of London, London, UK; 12 Centre for Personalised Medicine, Nuffield Department of Medicine, University of Oxford, UK; 13 NIHR Oxford Biomedical Research Centre, Oxford, UK

**Keywords:** Secondary genomic findings, 100,000 Genomes Project, healthcare outcomes, costs of disclosure

## Abstract

**Purpose:**

The UK 100,000 Genomes Project offered participants screening for additional findings (AFs) in genes associated with familial hypercholesterolaemia (FH) or hereditary cancer syndromes including breast/ovarian cancer (HBOC), Lynch, familial adenomatous polyposis, MYH-associated polyposis, multiple endocrine neoplasia, von Hippel-Lindau. Here we report disclosure processes, manifestation of AF-related disease, outcomes and costs.

**Methods:**

An observational study in an area representing one-fifth of England.

**Results:**

Data were collected from 89 adult AF recipients. At disclosure, among 57 recipients of a cancer predisposition-associated AF and 32 recipients of an FH-associated AF, 35% and 88% respectively had personal and/or family history evidence of AF-related disease. During post-disclosure investigations, four cancer-AF recipients had evidence of disease, including one medullary thyroid cancer. Six women with an HBOC AF, three women with a Lynch syndrome AF, and two individuals with a MEN AF elected for risk-reducing surgery. New hyperlipidaemia diagnoses were made in six FH-AF recipients, and treatment (re-)initiated for seven with prior hyperlipidaemia. Generating and disclosing AFs in this region cost £1.4m; £8,680 per clinically significant AF.

**Conclusion:**

Generation and disclosure of AFs identifies individuals with, and without personal or familial evidence of disease, and prompts appropriate clinical interventions. Results can inform policy towards secondary findings.

## Introduction

Genome sequencing has utility for understanding genetic contributions to rare disease and cancer(1,2) and its use in research and clinical settings has significantly increased in recent years. The scope of genome sequence analysis can technically be extended to include a search for variants associated with risks of future or asymptomatic disease, which may be unsuspected. Identified variants that are not pertinent to the presenting health condition have been termed incidental or, when intentionally sought, secondary findings. In 2013, the American College of Medical Genetics and Genomics (ACMG) proposed that a list of genes associated with conditions that are medically actionable before symptoms develop should be screened in individuals undergoing genome sequencing(3,4). Other professional groups do not recommend intentional clinical analysis of genes beyond those linked to the primary condition(5,6). Studies exploring attitudes of patients, health professionals, researchers and the public find broad support for the generation and return of actionable secondary findings(7). Identification of individuals at risk of associated diseases could inform surveillance for early disease detection and risk management, potentially saving lives and costly treatment of late-diagnosed disease. However, there is also potential for overdiagnosis, unwarranted medical intervention and anxiety, and justice arguments have been raised about offering ‘opportunistic’ screening to people already undergoing genome sequencing(8). A search and disclosure policy remains the subject of clinical and ethical debate(9,10), which has tended to focus on genome screening *per se*, with less attention paid to wider issues of clinical utility or the value and costs to patients and healthcare systems of extensive, recurrent clinical investigations and interventions to manage risk(11).

The UK 100,000 Genomes Project (100KGP), which began recruitment through the NHS in 2015, offered participants limited secondary findings, which Genomics England termed ‘additional findings’ (AFs), pathogenic and likely pathogenic (P/LP) variants in a number of genes associated with hereditary breast/ovarian cancer syndrome (HBOC, *BRCA1, BRCA2*); Lynch syndrome (*MLH1, MSH2, MSH6*); familial adenomatous polyposis (FAP, *APC*); MUTYH-associated polyposis (MAP, biallelic *MUTYH*); multiple endocrine neoplasia (MEN1, *MEN1* and MEN2, *RET*); von Hippel-Lindau syndrome (*VHL*); familial hypercholesterolaemia (FH; *LDLR, APOB, PCSK9, APOE* (p.Leu167del)). Around 1% of the UK population are thought to harbour a P/LP variant in one of the genes underlying breast/ovarian cancer predisposition, Lynch syndrome, and FH(12).

Identification of a pathogenic variant is not synonymous with a clinical diagnosis(13). While studies assessing genotype and phenotype in unselected biobank cohorts find considerable under-ascertainment of affected individuals, variant penetrance (the proportion of variant-carrying individuals who develop disease) is lower than in clinically ascertained families for a range of conditions(14), specifically FH(12,15–19); hereditary breast/ovarian cancer syndrome(12,17,18,20–23); and Lynch syndrome(12,17,18). While some biobank studies have reported on clinical outcomes of disclosing clinically actionable variants(16–24), there are few reports of communicating secondary findings in populations undergoing genome sequencing for diagnostic purposes(25). In their review, Sapp et al(25) found more evidence about disclosure practices than outcomes of secondary findings and concluded that evidence is limited regarding the prevalence of features consistent with specific secondary findings, healthcare use and behaviours, impacts on recipients, and cost-effectiveness. To address these questions in a real-world clinical setting, we undertook an observational study of participants receiving an AF from 100KGP in the UK NHS in one geographical area of England. We report variants identified and reported as AFs, disclosure processes, demographics and AF-related disease expression in recipients and their families, clinical investigations and interventions offered to assess and manage disease risk, and costs of identification, and disclosure. Consequent behaviours and psychosocial impacts on recipients were studied using qualitative methods and will be reported separately.

### Setting

The 100KGP recruited around 85,000 adults and children with undiagnosed rare disease or cancer through the UK NHS between 2015 and 2018(26). During recruitment, 92% of participants answered ‘yes’ to the offer of a search for AFs. Further details are in supplementary material. Disclosure consultations for individuals in the present study were held between November 2021 and October 2022.

## Methods

This study reports on generation of AFs, disclosure processes and outcomes in the Central and South Genomic Medicine Service (C&S GMS), one of seven NHS England alliances covering around one fifth of the population of England. The study was approved by South Central Berkshire B Research Ethics Committee (reference 21/SC/0254) and NHS Health Research Authority Confidentiality Advisory Group (reference 21/CAG/0160). An AF is defined as a confirmed P/LP variant not previously reported to the 100KGP participant in whom it was found.

### Data collection

A Patient Notification Document (PND; supplement) was designed by the study team and 100KGP Participant Panel Chair (JHW), informing participants of their right to opt out of the present study. Where clinical teams considered it appropriate, they sent the PND to adult participants after attendance at an AF disclosure appointment. Children in 100KGP were offered only a subset of AFs(27) and were not sent a PND. Data were collected relating to patients who were sent a PND and did not opt out after a minimum of two weeks. Case report forms were devised for each AF-associated condition with input from clinical teams, to collect demographic data, affected status with respect to primary condition, personal and family history, referrals for AF-indicated clinical investigation or care, risk management processes and outcomes. Data were collected from review of medical records (including but not limited to the disclosure consultation) held at the hospital site disclosing each patient’s AF, by the clinical or clinical research team. Online data collection meetings between the site teams and study team were held prior to and during data collection, and the first author visited sites to review data. Family history data collected were as reported by the AF recipient to their care team and were not verified. Post-disclosure healthcare data were collected by review of all data available at each site up to and including 31^st^ March 2023, a mean of 51.9 weeks (range 24-72.9) since AF disclosure. Variant data were obtained from clinical laboratories.

### Costs

**Figure 1:**
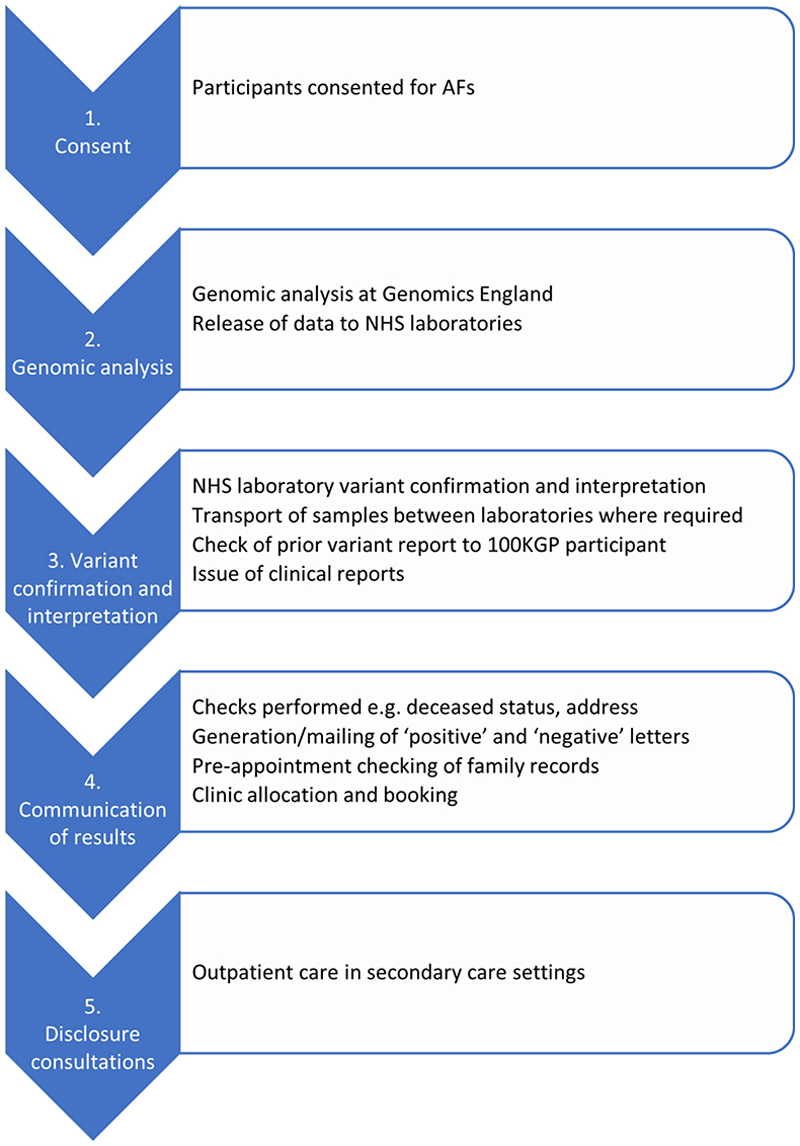
Sequential processes associated with AF generation and disclosure for which costs were estimated

In brief, costs associated with all pipeline processes (Figure 1) were calculated and combined to estimate the total cost of disclosing AFs in the C&S GMS. Costs were calculated from a healthcare provider perspective, from the initial consent process up to and including the return of AFs in outpatient appointments in secondary care. The costs of follow-up care (tests, interventions) occurring after the disclosure consultation, and family cascade health service use were not included. Data on resource use and unit costs were extracted from multiple data sources, including laboratory records, national pay scales and NHS reference cost databases. Base case values were identified for all parameters, and low/high values were specified for key potential cost drivers, for use in one-way sensitivity analysis. For step 5 in the costing process (disclosure consultations), data were only available for 89 of a total of 157 individuals with an AF. We therefore scaled up the total cost by 1.76 (157/89) to estimate disclosure-related healthcare costs across the whole population receiving an AF. A detailed description of the costing methods, parameters and data sources is provided in the supplementary materials. Costs were calculated per participant with an AF panel applied, per putative AF, and per individual with a true (disclosed) AF. One-way sensitivity analysis was undertaken for key potential cost drivers.

### Data analysis

To understand whether identification of an AF associated with cancer predisposition or FH differed according to recruitment arm (cancer or rare disease) of 100KGP(26), we used Fisher’s exact test for 2x2 tables to determine whether there was a difference in AF-relevant disease (evidenced by personal and/or family history) between patients with an AF associated with cancer predisposition or FH. Statistical significance was defined as p<0.05.

## Results

### AF variant analysis and report

**Figure 2:**
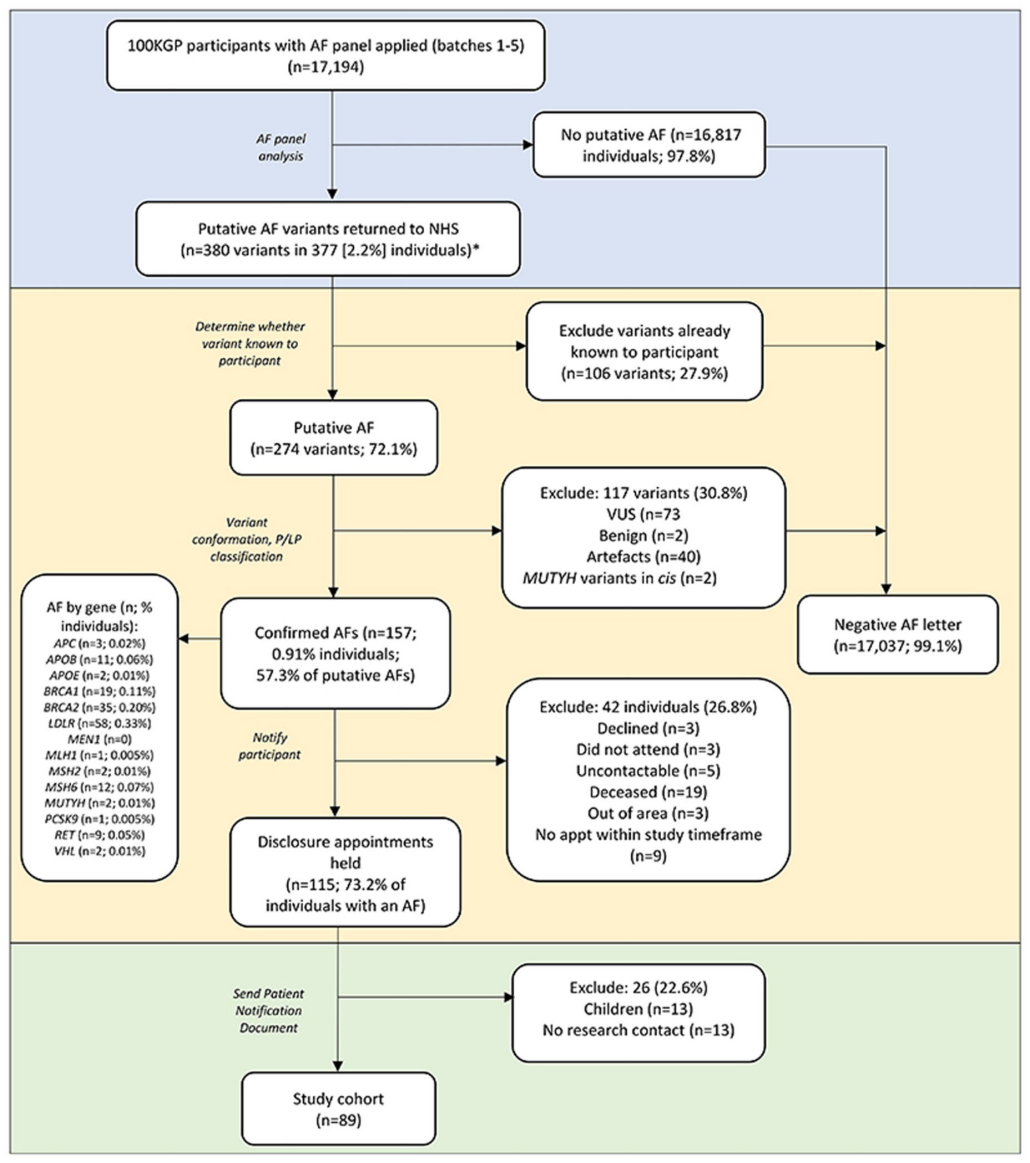
Flowchart showing 100KGP additional findings pipeline. Shown in blue are Genomics England activities; in yellow, NHS clinical laboratory or clinical services activities; in green, research activities. The number of variants or 100KGP participants at each stage is shown, including a gene-level breakdown of AFs identified. *Three individuals had two putative AFs; in each case one AF was reported and one variant removed after filtering.

Figure 2 and Table S1 show the process of AFs variant generation and handling through to disclosure and study cohort inclusion. Genomics England analysed an AF panel in genomes of 17,194 participants recruited to 100KGP in C&S GMS who elected for AFs and identified 380 variants (putative AFs) in 377 (2.2%) individuals, of which 106 variants (27.9% of putative AFs) had already been reported through standard of care testing, or primary 100KGP findings. Forty (10.5%) putative AFs were artefacts or unconfirmed, 73 (19.2%) were of uncertain significance (VUS) and two (0.5%) benign. Heterozygous *MUTYH* variants were found in two individuals in *cis*. These 117 variants (30.8% of all putative AFs) were not reported to clinical teams. Three individuals had two putative AFs; in each case one AF was reported and one variant removed after filtering.

### Disclosure

An AF was found in 157 (0.91%) 100KGP participants in C&S GMS in the study period, including 13 children and 21 now-deceased individuals (Table S1); a relative of two deceased individuals attended a disclosure appointment and received a PND. Patients were offered in-person or remote consultations to disclose their AF and discuss implications and proposed clinical management. Clinical teams were unable to contact five patients, and six did not engage with clinical contact or actively declined further information. Disclosing clinical specialists and processes varied by site and AF gene (Table 1). Some sites conducted a two-step disclosure process. In all trusts, AFs in cancer predisposition genes were disclosed by Clinical Genetics personnel, either clinical geneticists or genetic counsellors; AFs in FH genes were disclosed by specialist nurses either through Clinical Genetics, a bespoke nurse-led FH service, or a lipid clinic consultant. In the latter case, patients were clinically assessed and managed by the disclosing physician or referred to a local specialist service, unless already under the care of a lipid clinic. All other AF recipients were referred to specialists for clinical assessment and management.

### Participants

102 adult AF recipients had a disclosure consultation within the study time frame. For 13, clinical teams considered it inappropriate to send the PND. No individuals opted out. Data were collected from 89 AF recipients from 85 families who represent the study cohort. There were 67 unique variants in 11 genes. Mean recipient age was 46 years (range 23-83), and 39 (44%) were female. Ethnicity data were collected from medical records and stated as White British for 66 (74%). Thirty-seven (42%) individuals were affected with the condition for which they were recruited to 100KGP. For 59 (66%), no primary finding had been reported.

In the study cohort, a cancer predisposition gene AF was disclosed to 57 participants, 48 (84%) in the rare disease recruitment arm and nine (16%) in the cancer arm. An FH gene AF was disclosed to 32 participants, 28 (88%) in the rare disease arm and 4 (13%) in the cancer arm. Differences in prevalence of AF by gene and recruitment arm were not statistically significant (Table 1).

### Evidence of AF-related disease at disclosure

**Figure 3:**
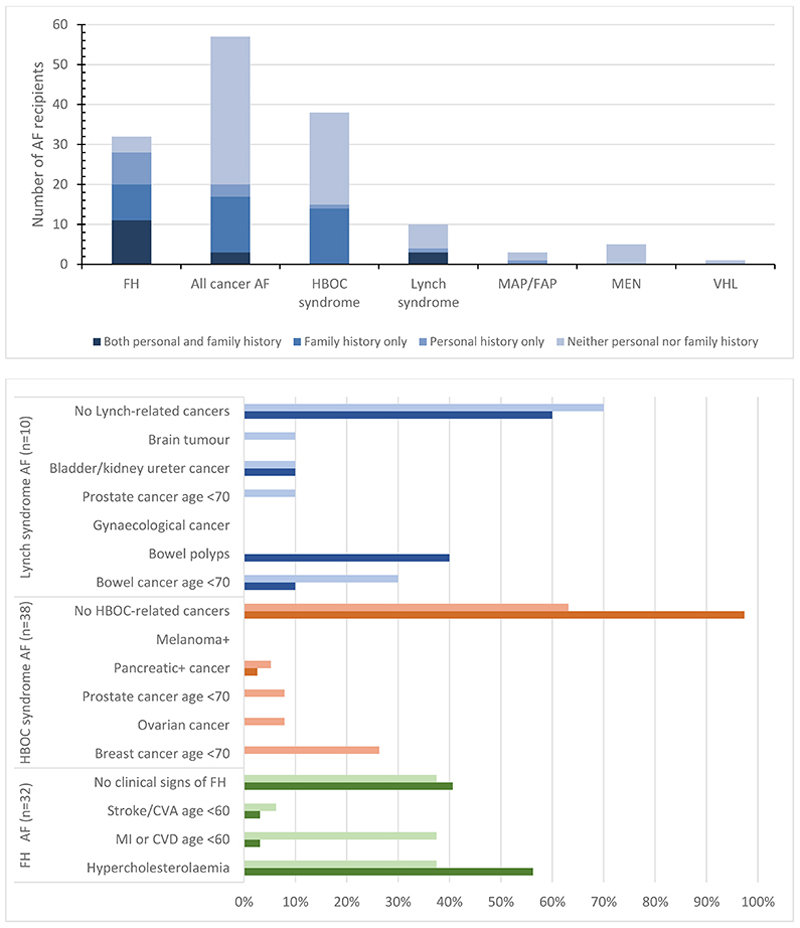
**a.** Numbers of AF recipients in the study cohort with a personal and/or family history of AF-related disease known at disclosure. **b.** Proportion of recipients of an AF in: FH-related gene (green bars); BRCA gene (orange bars); and Lynch syndrome (blue bars) with personal or family history of specific diagnoses or clinical signs of features consistent with the AF at disclosure. Darker and lighter shades represent personal and family history respectively. FH, familial hypercholesterolaemia; HBOC, hereditary breast and ovarian cancer; MAP, MUTYH-associated polyposis; FAP, familial adenomatous polyposis; MEN, multiple endocrine neoplasia; VHL, von Hippel-Lindau syndrome; MI, myocardial infarction; CVD, cardiovascular disease; CVA, cardiovascular accident; FH, familial hypercholesterolaemia. ^+^ *BRCA2* only

At disclosure, 20/57 (35%) and 28/32 (88%) recipients of an AF in a cancer-associated gene and FH-associated gene, respectively, had an apparent personal and/or family history potentially relevant to the AF (Table 1, Figure 3a,) as defined in Table S2. This difference is statistically significant (p=<0.001) and remains significant when including family history of diagnoses at unknown age or older age than would suggest a primarily monogenic cause. Since genotype information was not available for relatives except where stated, it is not possible to attribute relatives’ reported phenotypes definitively to the AF. Specific diagnoses or clinical findings noted in patient personal and family history for FH, hereditary breast/ovarian cancer syndrome and Lynch syndrome are shown in Figure 3b.

### FH

Among participants receiving an AF related to FH (n=32, age range 29-66, female n=12), 18 (56%) had a relevant personal history: 18 had a prior diagnosis of FH or hyperlipidaemia including one who had a cerebrovascular accident (CVA) aged in their thirties, and one a myocardial infarction (MI) aged in their forties. Two had possible achilles tenosynovitis, of whom one had known hyperlipidaemia. One person without known hyperlipidaemia had an abdominal aortic aneurysm. Eleven of these 18 also had a family history in a first-degree relative (FDR) or second-degree relative (SDR) of at least one FH-related concern including eight with a family history of hyperlipidaemia, six with premature cardiovascular disease (CVD) or MI, and one with a CVA.

Of 13 individuals without known personal history of FH or hyperlipidaemia, nine had a family history including at least one of hyperlipidaemia (n=4), premature MI/CVD (n=5), or CVA (n=1). Four individuals had either no known personal or family history (n=3) or reported a family history of a cardiovascular event at unknown age. Pre-disclosure low density lipoprotein-C (LDL-C) measurements were not available for most recipients or for any relatives and no family had a prior genetic diagnosis of FH, precluding a distinction between hyperlipidaemia and FH.

### Cancer predisposition

Among participants with a cancer predisposition gene AF (n=57, age range 23-83 years, female n=27), six (11%) had a personal history of cancer or clinical signs relevant to the AF including bowel polyps. Three of the six also had a relevant family history. Fourteen (25%) had only a family history and 37 (65%) had neither personal nor family history.

Thirty-eight participants received a *BRCA* AF (age range 23-69, female n=17). One had a personal history of *BRCA*-associated cancer, pancreatic acinar cell carcinoma diagnosed aged in their seventies and for which they were recruited to the 100KGP cancer arm; this individual’s mother was diagnosed with breast cancer aged in her seventies, and child with bile duct cancer aged in their forties (the *BRCA2* variant was not reported as a primary finding). For three individuals without personal history of cancer the variant was already known in recipients’ families, having been identified during standard clinical care based on family history. The AF recipient in one of these families was aware of the familial variant and had actively deferred pre-symptomatic testing. Of the remaining 34, 11 had a family history suspicious for HBOC (Table S2), including seven with a family history of breast cancer. Of the seven, two also had an FDR diagnosed with prostate cancer (one aged in their fifties and sixties, respectively). Among the remaining four families, two had an FDR diagnosed with ovarian cancer, one an FDR with pancreatic cancer diagnosed age 74, and one with a relative diagnosed with prostate cancer aged in their fifties. Sixteen individuals (42.1%) reported no BRCA-related personal or family history. A further six individuals reported some family history of *BRCA*-related cancer diagnosed in elderly individuals or at an unknown age, or uncertain diagnosis; we did not classify these families as having a positive history of HBOC. Family history information was unavailable for one individual.

Ten participants received a Lynch syndrome-associated AF (female n=5, age range 25-92). Four (40%) had a relevant personal history: one bowel mucinous adenocarcinoma (for which they were recruited to the cancer arm of 100KGP; the AF was not reported as a primary finding) and prostate adenocarcinoma *in situ* both diagnosed in their sixties, and a history of bowel polyps. Three relatives of that individual had bowel cancer aged in their seventies, and an adult child had kidney cancer. A further individual had papillary transitional cell carcinoma of the bladder/ureter and bowel polyps aged in their eighties and an FDR diagnosed with bowel cancer aged in their forties. Two further individuals had a history of bowel polyps: in the family of one, two relatives had a history of bowel cancer, three of brain tumour and two of prostate cancer. Six individuals had no suspicious family history, although two reported some family history diagnosed in elderly individuals or at an unknown age.

Two participants received an *APC* AF; neither had relevant personal or family history. The one individual with biallelic *MUTYH* (homozygous) had a personal history of bowel polyps below age 35 and reported no family history. Five participants had a *RET* AF and one a VHL AF; none reported personal or family history.

### Clinical investigations and outcomes

Outcomes after return of AFs are shown in Table 2. For recipients of an FH-associated AF (n=32), a mean of 52.3 weeks (range 27.3–72.0) had elapsed between disclosure appointment and final data interrogation. A lipid screen was arranged for 28 individuals. Of the 14 (44%) not known to have hyperlipidaemia at disclosure, outcomes data were available for six who all began lipid-lowering therapy. Two had total cholesterol measurements below 6 mmol/L and statin therapy was initiated due to borderline total cholesterol or raised LDL-C. Of 18 (56%) individuals in whom hyperlipidaemia was diagnosed before AF disclosure, seven were not taking lipid-lowering medication either because no prescription had been made, or the individual had discontinued treatment. AF identification in individuals with prior hyperlipidaemia prompted a change in management for 17: (re-)introduction of lipid-lowering therapy, initially statin (n=13), supplemented with ezetimibe (n=1), or statin replaced by a PCSK9 inhibitor together with ezetimibe (n=1), or increased dose (n=2). Ongoing care was arranged or continued through a lipid clinic or other physician for 30 individuals.

Among recipients of an AF in a cancer-predisposition gene (n=57, 55 living), a mean of 51.7 weeks (range 24–72.9) had elapsed between disclosure appointment and final data interrogation. Some clinical outcomes data were available for 22; four had a relevant post-disclosure diagnosis.

All 16 age-eligible female recipients of a *BRCA1/2* gene AF were referred for breast imaging. Age-eligible male *BRCA1/2* AF recipients (n=17) were recommended to discuss prostate cancer risk/screening with their GP or referred to urology. One man sought a mammogram. Of 17 women with a *BRCA1/2* AF (age range 24-69), ten were referred for discussion of risk-reducing mastectomy (RRM). Of four for whom outcomes data were available, two elected for surgery. Six women elected against RRM referral at AF disclosure. Ten women were referred for discussion of risk-reducing bilateral salpingo-oophorectomy (RRBSO). Of five for whom outcomes data were available, four elected for surgery; three for conventional RRBSO, and one had early salpingectomy with delayed oophorectomy as part of the PROTECTOR study(28). A *BRCA1* variant disclosed to one individual (without prior personal or family history of *BRCA*-related cancer) was re-classified from LP to VUS during the study period after national variant discussions. The patient had attended consultations with breast and gynaecology surgery teams but had not made surgical decisions.

All nine living recipients of a Lynch syndrome AF were referred for bowel screening or to a Lynch syndrome MDT clinic. Colonoscopy results were available for two individuals (aged in their 50s). One small polyp was found in both, one of whom had a previous bowel polyp removal. Seven individuals were referred to their GP for a *Helicobacter pylori* test (no outcomes data available). Three commenced daily aspirin. Three women were referred to gynaecology and all elected for risk-reducing hysterectomy and RRBSO. The single *MSH2* AF recipient was referred for kidney scans in addition to bowel screening (no outcomes data available).

Both recipients of an *APC* gene AF were referred for colonoscopy and endoscopy. Outcome data are available for one individual aged in 40s with no prior personal or family history. Four bowel polyps (two sessile, two adenomatous) were found. Gastroscopy was normal. The individual with biallelic *MUTYH* AF was referred for bowel screening (no outcomes data available). All five *RET* gene AF recipients received some screening including four for thyroid ultrasound scans and four for biochemical tests. One individual aged in their 40s with AF NM_020975.6(RET):c.2410G>A (p.Val804Met) without prior personal or family history of MEN-related disease was initially found to have raised calcitonin and underwent total thyroidectomy; a medullary thyroid carcinoma was detected. A second individual underwent risk-reducing thyroidectomy following a thyroid ultrasound scan showing bilateral nodules. The recipient of a *VHL* AF attended a VHL clinic, an ophthalmology clinic and had an abdominal MRI scan with normal findings.

For individuals with an AF in genes associated with FAP, MAP, and VHL, no risk management procedures were documented during the study period.

### Costs of disclosure

Costs were calculated or estimated for the processes shown in Figure 1 and supplement. The mean number of disclosure-related outpatient episodes was 1.35 and the mean cost of outpatient care was £555 per recipient in the study cohort (Table 3). Participants with a cancer-related AF had more disclosure outpatient episodes (1.54 vs 1.00) and accrued greater outpatient care costs (£714 vs £270) than participants with an FH-associated AF. Cost differences by trust and gender reflected differences in episode coding and case mix, as well as differing proportions of episodes that were consultant-led. The total cost of generating and disclosing AFs in the C&S GMS is £1.4m (Table 4). This represents a cost of £79 per participant in whose sample an AF panel was applied, £3,615 per participant with a putative AF, and £8,680 per disclosed AF. The most expensive component is genomic analysis (£1,065,261). One-way sensitivity analysis indicated that most parameter variations had no effect on the study results. The one exception was the cost of the Genomics England AFs pipeline: when this increased from £56 per genome to £84 per genome, the cost per new AF identified increased from £8,680 to £11,746. When this cost reduced from £56 per genome to £28 per genome, the cost per new AF identified decreased from £8,680 to £5,613.

## Discussion

This is the first report of identification and disclosure through the NHS of 100KGP AFs, clinically actionable secondary findings in a limited set of genes associated with cancer predisposition and FH, to adult participants. This observational study addresses several aspects of clinical utility of genomic testing(29) including diagnostic thinking, therapeutic management, patient health outcomes, and economic costs. A clinically actionable AF was reported in 0.91% of 17,194 100KGP participants who elected for AFs screening. From data extracted from medical records for 89 adults who attended an AF disclosure consultation, 48 AF recipients (54%) had a relevant personal and/or family history at disclosure. Personal and family histories were significantly more common in recipients of an FH-associated AF than a cancer predisposition-associated AF, in line with studies investigating disease evidence in population studies(12,14,17,18). Cancer-related AF disclosure was managed through Clinical Genetics, and specialist referrals made for clinical investigation and care. Disclosure of FH-related AF was managed either via a lipid clinic consultant who also co-ordinated management, or via specialist FH nurses. Clinical care arranged for AF recipients was consistent with UK recommendations irrespective of personal and family history, and most participants engaged with recommended screening. In ten individuals for whom outcomes data were available, a clinical diagnosis of AF-related disease was made during post-disclosure clinical investigations. Overall, the AFs analysis and disclosure process cost £79 per participant, and £8,680 per individual to whom an AF was disclosed. The overall cost of generating and disclosing AFs across the C&S GMS was £1.4m.

One *BRCA* variant, detected in a woman in her 30s without family history of cancer, was re-classified from LP to variant of uncertain significance during the study period. This case highlights a potential significant harm of opportunistic screening. Although genetic counselling can aim to support nuanced decision making around risk management, it may not be possible to allay patient uncertainty and anxiety before and after re-classification, particularly when risk management strategies are life-altering and irreversible. Our study includes three individuals in whose family there was a clinically reported variant for which the AF recipient had not personally undergone predictive testing. One individual had actively chosen to defer testing for the familial (*BRCA*) variant until around the time at which breast screening would begin, highlighting the need for effective informed consent and illustrating potential psychological harms to individuals and families which may be exacerbated by a considerable time gap between consent and disclosure.

Our findings suggest that opportunistic screening for FH would identify many individuals with FH who are not under medical care, leading to initiation of or change in lipid-lowering therapy. The finding that seven individuals had a prior diagnosis of hyperlipidaemia but were not taking lipid-lowering medication highlights the need for increased primary care and patient awareness of FH. In UK Biobank, LDL-C levels were significantly higher among individuals with a heterozygous P/LP FH variant, who had a three-fold risk of developing atherothrombotic cardiovascular disease compared with individuals who did not have a P/LP FH variant(12). US population prevalence of hyperlipidaemia among individuals with a heterozygous P/LP FH variant is 87%(19). FH is underdiagnosed and undertreated in most countries(30); NHS England estimate that less than 8% of affected people are currently identified(31). Most individuals can be managed in primary care at low cost after an initial lipid clinic assessment, and LDL-C can be routinely measured allowing phenotype-guided treatment and monitoring of efficacy, and therapy implemented irrespective of age. Genetic diagnosis is valuable for risk stratification and family cascade testing(32), and our data show that a genetic diagnosis can prompt changes in clinical care regardless of prior clinical diagnosis.

Regarding opportunistic screening for cancer predisposition, our data are less compelling; a small minority of individuals with a heterozygous P/LP variant had personal evidence of relevant disease. However, evidence of AF-related disease was found during post-disclosure investigations, highlighting the value of generating and disclosing AFs. For *BRCA*-related cancer in women and Lynch syndrome-related gynaecological cancer predisposition, no reliable intermediate biochemical or clinical measures of disease manifestation are available, and in our cohort, several unaffected women for whom data are available elected for risk-reducing surgery. A low rate of cancer diagnosis at disclosure in our cohort (age range 21-92 for cancer AFs) does not preclude increased risk of cancer at older age. Indeed, in an older cohort, the prevalence of relevant cancer was significantly increased among individuals with a heterozygous P/LP variant: 4.11-fold for females with a heterozygous P/LP *BRCA1/2* variant and 12.77-fold for individuals with a P/LP Lynch syndrome variant(12). Family history is limited as a means of identifying individuals with a heterozygous P/LP variant: a large proportion of individuals with a heterozygous P/LP variant (75% for HBOC, 63% for Lynch syndrome, 34% for FH) had no family history of relevant disease in an FDR(12) or would not qualify for genetic testing under relevant guidelines (67% for HBOC, 77% for Lynch, 86% for FH(17)). In another biobank study, 34% of individuals with a heterozygous P/LP *BRCA1/2* variant would not meet testing criteria(20).

The 100KGP AFs genes(27) are a subset of the ACMG secondary findings gene list(3,33), and do not include genes associated with inherited cardiac conditions (ICC), which account for a large proportion of all ACMG secondary findings(34). Penetrance of ICC gene variants is incomplete: for two of these prevalent disorders, hypertrophic cardiomyopathy and dilated cardiomyopathy, variant penetrance in UK Biobank is 23% and 35% respectively(35). Our earlier small studies report on the complexities of secondary findings in ICC(36,37). The ACMG continue to revise and expand their secondary findings gene list(33), notwithstanding the need to accumulate evidence of clinical utility(3).

We have presented information on the costs of AFs generation and disclosure but did not conduct a formal economic evaluation due to the narrow scope of our analysis. The estimated cost per true AF identified in our study population was £8,680. Determining the cost-effectiveness of a policy of offering AFs, including whether this falls below the National Institute for Health and Care Excellence cost-effectiveness threshold of £20,000-£30,000 per unit of effectiveness gained(41), will require studies expanding the analytical perspective to capture all costs and consequences, including short and long-term cost implications and impacts of returning AFs on life expectancy and quality of life.

Our cost estimates are broadly in line with the limited literature. For individuals in the USA receiving secondary findings from the ACMG-recommended list, the mean cost of follow-up medical actions per finding up to one year post-disclosure was $128-$421, depending on medical action responses(38). In a modelling study evaluating the resource implications of returning secondary findings in Australia, the cost per individual was $430, and the cost per clinically significant finding $4,349(39). Population genomic sequencing in the USA for a panel of high-evidence genes associated with FH, HBOC and Lynch syndrome was judged likely cost-effective when compared with US cost-effectiveness thresholds, at $68,000 per QALY gained(40). However, an earlier US modelling study reported that returning secondary findings is unlikely to be cost-effective for generally healthy individuals(41).

We have previously reported expert views that an approach to opportunistic screening should be at variant-level(9), and this view is supported by evidence that penetrance is heterogeneous even within the same disease gene(14,19). Since monogenic disease expression is modified by common genetic variation(42–45), incorporating polygenic risk scores (PRS) with screening for monogenic variants might in the future increase the accuracy of risk estimation and be used to tailor genetic counselling and risk management. However, PRS are based on genome-wide association studies, in which the majority of participants are of European descent, meaning that PRS are not generalizable to globally diverse populations(46).

Opportunistic genomic screening is distinct from population screening, and recommendations to report secondary findings are not necessarily an endorsement of population screening in a public health context(47). The ACMG propose that DNA-based risk detection should be evidence-based and comply with health screening criteria(48), and UK guidance criteria for population screening programmes are based on the same principles(49). One criterion is that the ‘natural history’ of a condition proposed for screening should be understood, including penetrance and age of onset in individuals with a heterozygous P/LP variant; such data remain limited. Health equity is imperative for a genomic screening policy(50), and implementation should consider design to benefit the whole population(13). A targeted approach - considering age of commencement of screening and risk management for a given condition - would offer greater population benefits than opportunistic genomic screening, while minimising risk of psychological harms that might result from disclosing a disease-predisposing variant several years before screening would be offered. Given the reduced costs of genetic testing (a bespoke gene panel may be more cost-effective than genome sequencing), population genetic screening could re-focus resources at an earlier stage in disease development, with advantages for individuals and health systems(15,51,52). Implementation of a targeted approach would require separate considerations for cancer predisposition and FH, and while a disease-specific approach would inevitably place a burden on health services, cancer- and FH-risk are managed by appropriate care specialisms. Maximising the utility of population screening while minimising psychological harms will require genomic counselling to promote communication to relevant family members, psychological support and referral for appropriate risk assessment and management, and care in delivery to minority groups. The current under-representation of individuals without recent north European ancestry in genomic datasets(46) presents a challenge to equitable genomic healthcare. Workforce planning and education to support delivery of preventative healthcare requires a long-term outlook.

## Limitations

This study presents data from a real-world clinical situation and is limited by relatively small numbers of AF recipients and limited outcomes data available. In many cases specialist investigations took place at non-participating hospitals or after the study timeframe, and we are unable to report on pursual of referrals. Including family history of potentially relevant disease is likely to overestimate disease occurring due to the variant identified as an AF, since monogenic predisposition to cancer and FH (or hyperlipidaemia) represents a small proportion of total disease prevalence and in ungenotyped relatives, monogenic disease cannot be distinguished from multifactorial disease. We did not seek to verify patient-reported family history data.

Some limitations should be noted related to the cost analysis. First, we assumed all participants were consented individually but some may have been consented as a family group, slightly overestimating consent costs. Second, as disclosure-related secondary care resource use data were only available for a subset (89 of 157 participants with an AF), we scaled up this cost to estimate secondary care costs related to AFs disclosure across the population (n=157), potentially overestimating costs in this category. Third, data were not available for most of the resource use items included in the analysis to facilitate the extension of our analysis to consider the uncertainty surrounding our results using probabilistic sensitivity analysis. However, one-way sensitivity analysis suggests that there is one major cost driver: the cost of the Genomics England AFs pipeline. Fourth, this was an observational study with no comparator group. Future studies comparing populations who receive AFs with those who do not could allow more robust conclusions to be drawn about the value of returning AFs.

The health economic analysis performed is restricted to processes of generation and disclosure of AFs and does not include subsequent tests or interventions. Further research is required to understand longer-term health outcomes following disclosure, the value of providing care to AF recipients over the lifespan, impact on life expectancy, personal utility, and the extent to which AFs disclosure led to family cascade testing. Meaningful costing of follow-up care would require longer-term capture of sequential investigations, interventions, and family testing.

## Conclusions

This study addresses several aspects of the clinical utility of secondary findings in selected genes associated with cancer predisposition and FH, including correlation with phenotype, clinical care interventions, patient health outcomes, and costs of generation and disclosure. Findings show that disclosing clinically significant secondary genomic findings in these genes identifies individuals with, or at risk of associated disease, and can prompt appropriate clinical interventions. Evidence of relevant disease was present in a significantly greater number of recipients of an FH-associated AF than in recipients of a cancer-associated AF. Questions of resourcing and equitable implementation of generating potentially disease-associated genomic findings in clinically unascertained populations, either as secondary findings or in a population screening context, require improved understanding of the natural history of these health conditions and long-term outcomes.

## Supplementary Material

Supplementary Material

## Figures and Tables

**Table 1:** Participant demographics, AF gene, recruitment arm, primary condition status and result category, personal and family history of AF-related disease, disclosure process

	Cancer										FH							
Gene	*BRCA1*	*BRCA2*	*MSH2*	*MSH6*	*MUTYH*	*APC*	*RET*	*VHL*	Cancer AF	%	*LDLR*	*APOB*	*APOE* ^a^	FH AF	%	Total	%	P value
AF variants in study cohort																		
AF	12	26	1	9	1	2	5	1	57	64.0%	26	5	1	32	36.0%	89		
Unique variants	12	19	1	8	1	2	3	1	47		18	1	1	20		67		
Unique families	12	24	1	9	1	2	4	1	54		25	5	1	31		85		
Demographics																		
Female	4	13	1	4	-	2	3	-	27	47.4%	7	4	1	12	37.5%	39	43.8%	
Age range	31-69	23-60	50	29-56	-	42-50	59-83	-	23-83		39-66	29-65	32	29-66		23-83		
Mean age (years)	43.3	38.5	50	44.8	-	46	72	-	44.8		51.4	49.8	32	49.1		46.1		
Male	8	13	-	5	1	-	2	1	30	52.6%	19	1		20	62.5%	50	56.2%	
Age range	51-81	21-82	-	41-92	36	-	23-46	64	21-92		24-69	44	-	24-69		21-92		
Mean age (years)	63.4	48.9	-	62.2	36	-	34.5	64	54.1		47.9	44	-	47.8		51.6		
Ethnicity																		
White British	8	21	1	7	1	2	4	1	45	78.9%	16	5	0	21	65.6%	66	74.2%	
White, other White background	0	1	0	0	0	0	0	0	1	1.8%	1	0	0	1	3.1%	2	2.2%	
British Asian, Indian	3	0	0	0	0	0	0	0	3	5.3%	0	0	0	0	0.0%	3	3.4%	
Black British, Afro-caribbean	0	1	0	0	0	0	0	0	1	1.8%	1	0	0	1	3.1%	2	2.2%	
Black, other Black background	0	0	0	0	0	0	0	0	0	0.0%	1	0	0	1	3.1%	1	1.1%	
Mixed, White and Black Caribbean	0	0	0	1	0	0	0	0	1	1.8%	0	0	0	0	0.0%	1	1.1%	
Mixed, other mixed background	0	1	0	0	0	0	0	0	1	1.8%	1	0	0	1	3.1%	2	2.2%	
Chinese	0	0	0	0	0	0	0	0	0	0.0%	1	0	0	1	3.1%	1	1.1%	
Hong Kongese	0	0	0	0	0	0	0	0	0	0.0%	1	0	0	1	3.1%	1	1.1%	
Not stated	1	2	0	1	0	0	1	0	5	8.8%	4	0	1	5	15.6%	10	11.2%	
100KGP recruitment arm																		
Rare disease	9	24	1	7	0	2	4	1	48	84.2%	22	5	1	28	87.5%	76	85.4%	0.76
Cancer	3	2	0	2	1	0	1	0	9	15.8%	4	0	0	4	12.5%	13	14.6%	
100KGP primary condition status																		
Proband/affected	7	10	0	3	1	0	3	1	25	43.9%	10	2	0	12	37.5%	37	41.6%	
Unaffected	5	16	1	6	0	2	2	0	32	56.1%	16	3	1	20	62.5%	52	58.4%	
100KGP primary condition result																		
Likely cause identified	2	5	0	2	0	0	0	1	10	17.5%	7	2	0	9	28.1%	19	21.3%	
VUS/uncertain result	2	2	0	0	0	1	2	0	7	12.3%	1	3	0	4	12.5%	11	12.4%	
No cause identified	8	19	1	7	1	1	3	0	40	70.2%	18	0	1	19	59.4%	59	66.3%	
Evidence of AF-related disease																		
Only personal history	0	1	1	0	1	0	0	0	3	5.3%	7	1	0	8	25.0%	11	12.4%	
Only family history	5	9	0	0	0	0	0	0	14	24.6%	5	3	1	9	28.1%	23	25.8%	
Both personal and family history	0	0	0	3	0	0	0	0	3	5.3%	10	1	0	11	34.4%	14	15.7%	
Personal and/or family	5 (42%)	10 (38%)	1 (100%)	3 (50%)	1 (100%)	0 (0%)	0 (0%)	0 (0%)	20	35.1%	22 (85%)	5 (100%)	1 (100%)	28	87.5%	48	53.9%	<0.001
Neither personal nor family history	7	16	0	6	0	2	5	1	37	64.9%	4	0	0	4	12.5%	41	46.1%	
Disclosure process																		
Initial AF letter	12	26	1	9	1	2	5	1	57	100.0%	26	5	1	32	100.0%	89	100.0%	
Pre-disclosure appt phone call	6	12	0	7	1	0	2	0	22	38.6%	0	0	0	0	0.0%	22	24.7%	
AF disclosure appointments held by:																		
Consultant Geneticist	4	3	0	3	0	1	4	1	16	28.1%	-	-	***-***	0	-	16	18.0%	
Geneticist (Specialist Registrar)	0	0	0	0	0	0	1	0	1	1.8%	-	-	***-***	0	-	1	1.1%	
Consultant Genetic Counsellor	1	5	1	3	0	1	0	0	11	19.3%	-	-		0	-	11	12.4%	
Principal Genetic Counsellor	2	3	0	0	0	0	0	0	5	8.8%	***-***	-	***-***	0	-	5	5.6%	
Genetic Counsellor	4	14	0	3	0	0	0	0	21	36.8%	-	-		0	-	21	23.6%	
Trainee Genetic Counsellor	1	1	0	0	1	0	0	0	3	5.3%	-	-	-	0	-	3	3.4%	
Lipid Consultant	-	-	-	-	-	-	-	-	0	-	12	2	0	14	43.8%	14	15.7%	
FH Nurse Specialist	-	-	-	-	-	-	-	-	0	-	14	3	1	18	56.3%	18	20.2%	

No individuals in cohort with variant in *MEN1, MLH1*, or *PCSK9* VUS/uncertain result: variant of uncertain significance or uncertain explanation for phenotype

a *APOE* c.500_502 (p.Leu167del) only included in AF panel

**Table 2:** Post-disclosure risk assessment and risk management procedure referrals and outcomes

Hereditary breast and ovarian cancer syndrome n=38; living n=37; female n=17; mean (range) follow up weeks: 52.2 (24-72.9)				
Risk assessment	Referred	Attended (no data)	Normal outcome (no data)	New AF-related cancer diagnosis (no data)
VHR breast screening (mammogram or breast MRI)	16	5 (11)	5	0
Mammogram (symptomatic)	1	1	1	0
Prostate screening (GP or Urologist)	17	5 (12)	(17)	(17)
Post-disclosure genetic counselling	15	15	-	-
**Risk management**	**Referred**	**Attended (no data)**	**Decision to proceed**	**New AF-related cancer** **diagnosis**
RR breast surgery	10	4 (6)	2	0
RR ovarian surgery	10	5 (5)	4	0
**Lynch syndrome n=10; living n=9; female n=5; mean (range) follow up weeks: 48 (29.4-71.1)**				
**Risk assessment**	**Referred**	**Attended (no data)**	**Polyps found**	**New AF-related cancer** **diagnosis**
Colonoscopy (or Lynch MDTclinic)	9	2 (7)	2	0
Post-disclosure genetic counselling	2	2	-	
**Risk management**	**Referred**	**Attended (no data)**	**Decision to proceed**	**New AF-related cancer** **diagnosis**
Aspirin (GP prescription)	6	4 (2)	3	NA
*H. pylori* test (GP)	7	0 (7)	NA	NA
RR hysterectomy	3	3	3	0
**Familial adenomatous polyposis n=2; MUTYH-associated polyposis n=1; mean (range) follow up weeks: 49.8 (40.4-67.1)**				
**Risk assessment**	**Referred**	**Attended (no data)**	**Polyps found**	**New AF-related cancer** **diagnosis**
Colonoscopy	3	1 (2)	1	0
Endoscopy	2	1 (1)	0	0
Post-disclosure genetic counselling	2	2	-	-
**Multiple endocrine neoplasia; n=5; mean (range) follow up weeks: 53.3 (28.4-60.1)**				
**Risk assessment**	**Referred**	**Attended (no data)**	**Normal outcome**	**New AF-related cancer** **diagnosis**
Thyroid USS	4	2 (2)	1	0
Biochemical tests	4	3 (1)	2	1
Abdominal MRI	1	0 (1)	-	-
Post-disclosure genetic counselling	2	2	»»	
**Risk management**	**Referred**	**Attended**	**Surgery**	**New AF-related cancer** **diagnosis**
Thyroidectomy	2	2	2	1
**von Hippel-Lindau syndrome n=1; follow up weeks: 63**				
**Risk assessment**	**Referred**	**Attended (no data)**	**Normal outcome**	**New AF-related cancer** **diagnosis**
VHL clinic	1	1	1	0
Ophthalmology screening	1	1	1	0
Abdominal MRI	1	1	1	0
Post-disclosure genetic counselling	0	-	-	-
**Familial hypercholesterolaemia n=32; mean (range) follow up weeks: 52.3 (27.3-72)**				
**Risk assessment**	**Referred** **(no data)**	**Attended (no data)**	**Normal outcome**	**New AF-related diagnosis**
Lipid screen	18 (10)	10 (7)^a^	0	6 (of 6)
Post-disclosure genetic counselling	7	7		-
	**New referral**	**Attended (no data)**	**Continue existing plan**	
Lipid clinic	28	10 (17)^a^	4	
**Risk management**	**Begin therapy** **(no data)**	**Increase dose**	**Additional medicine**	**No change**
Medication	8(18)	2	2	4

**Table 3:** AF disclosure secondary care resource use and costs per AF recipient

Participants	Sample size	Outpatient episodes per participant^a^				Total cost per participant			
		Mean	SD	Minimum	Maximum	Mean	SD	Minimum	Maximum
All participants	89	1.35	0.48	1	2	£554.63	£297.13	£108.57	£1388.72
Type of additional finding									
Cancer	57	1.54	0.50	1	2	£714.25	£240.52	£400.91	£1388.72
Familial hypercholesterolemia	32	1.00	-	1	1	£270.33	£125.87	£108.57	£437.63
Gender									
Male	50	1.32	0.47	1	2	£538.03	£283.29	£108.57	£1187.10
Female	39	1.38	0.49	1	2	£575.92	£316.46	£108.57	£1388.72
Type of additional finding, by gender									
Cancer – female	27	1.56	0.51	1	2	£725.60	£252.86	£400.91	£1388.72
Cancer – male	30	1.53	0.51	1	2	£704.02	£232.72	£433.14	£1187.10
Familial hypercholesterolemia – female	12	1.00	-	1	1	£239.12	£126.89	£108.57	£437.63
Familial hypercholesterolemia – male	20	1.00	-	1	1	£289.05	£124.68	£108.57	£437.63
Trust									
Birmingham Women’s and Children’s Hospital	34	1.50	0.51	1	2	£695.84	£215.46	£437.63	£893.65
Oxford University Hospitals NHS Foundation Trust	26	1.42	0.50	1	2	£551.62	£310.83	£290.27	£1187.10
University Hospitals Birmingham	10	1.00	-	1	1	£108.57	-	£108.57	£108.57
University Hospitals Southampton	19	1.16	0.37	1	2	£540.84	£252.82	£400.91	£1388.72

SD = standard deviation.

a An episode is defined as a single outpatient appointment

**Table 4:** Overall cost of AFs generation and disclosure process in the C&S GMS

Process	Cost across the C&S GMS
1. Consent	£85,981
2. Genomic analysis	£1,065,261
3. Variant confirmation and interpretation	£79,773
4. Communication of results	£44,594
5. Disclosure consultations ^a^	£87,078
**TOTAL**	**£1,362,687**
	
Cost per participant with AF panel applied (n=17,194)	£79
Cost per participant with a putative AF (n=377)	£3,615
Cost per new AF identified (n=157)	£8,680

AF = additional finding; C&S GMS = Central and South Genomic Medicine Service.

a Disclosure-related secondary care resource use data were available for 89 of the 157 participants who received a positive AF letter, with a total cost of £49,362 (mean cost £554.63). This mean cost was applied for the 68 participants for whom disclosure-related secondary care resource use data were not available, giving a revised total cost for process 5 of £87,078

## Data Availability

Because of the sensitive nature of the data collected for this study, requests to access the datasets from qualified researchers trained in human subject confidentiality protocols may be sent to the University of Oxford via the corresponding author at liz.ormondroyd@cardiov.ox.ac.uk.
