# Supplementary material for: Secondary (additional) findings from the 100,000 Genomes Project: disease manifestation, healthcare outcomes and costs of disclosure
Source: Genet Med. Author manuscript; Available in PMC 2025 Jul 17. (PMC7617895; doi:10.1016/j.gim.2023.101051)
Supplement: Supplementary Material [file EMS206626-supplement-Supplementary_Material.pdf]

Supplementary materials

Table S1. Putative AF variants called by initial bioinformatic pipeline, all potentially pathogenic variants in AF genes, and prevalence of AF in 17,194 participant genomes in C&S GMS

Table S2. Criteria for inclusion of symptoms and clinical signs of AF-related disease in personal and family history

Setting

Cost calculations

Table S3 Parameters and data sources used in cost calculations

Table S4. One-way sensitivity analysis results

UK risk assessment and management recommendations for individuals with a heterozygous pathogenic variant

Patient notification document (PND)

Table S1. Putative AF variants called by initial bioinformatic pipeline, all potentially pathogenic variants in AF genes, and prevalence of AF in 17,194 participant genomes in C&S GMS

Gene	Putative AF	Variant already known	Artefact/ unconfirmed	VUS	Benign	MUTYH in cis	Potentially pathogenic variants identified^	AF variants	AF prevalence
APC	8	4	1	0	0	0	7	3	0.02%
APOB	29	2	0	16	0	0	13	11	0.06%
APOE	7	5	0	0	0	0	7	2	0.01%
BRCA1	34	13	1	1	0	0	32	19	0.11%
BRCA2	75	33	2	4	1	0	68	35	0.20%
LDLR	113	10	10	35	0	0	68	58	0.34%
MEN1	4	3	0	1	0	0	3	0	0.00%
MLH1	14	5	7	0	1	0	6	1	0.01%
MSH2	29	6	18	3	0	0	8	2	0.01%
MSH6	35	16	1	6	0	0	28	12	0.07%
MUTYH	6	2	0	0	0	2	4	2	0.01%
PCSK9	4	0	0	3	0	0	1	1	0.01%
RET	15	6	0	0	0	0	15	9	0.05%
VHL	7	1	0	4	0	0	3	2	0.01%
**Total**	380	106	40	73	2	2	263	157	0.91%
**All FH**							89	72	0.42%
**All cancer**							174	85	0.49%

^includes variants previously known to 100KGP participant and previously not known (AFs)

Table S2. Criteria for inclusion of symptoms and clinical signs of AF-related disease in personal and family history[1]

Condition (genes)	Personal history	Family history
FH<br>(*LDLR, APOB, APOE, PCSK9*)	One or more of:<br>• FH or hyperlipidaemia (or "raised/high/borderline raised" cholesterol)<br>• Myocardial infarction<br>• Coronary artery disease<br>• Atherosclerosis<br>• Achilles tenosynovitis$^{\Delta}$<br>• Xanthoma xanthelasma$^{\Delta}$<br>• Corneal arcus$^{\Delta}$<br><br>$^{\Delta}$not included if present in isolation	One or more of:<br>• FH or hyperlipidaemia (or "raised/high/borderline raised" cholesterol)<br>• Myocardial infarction age <60<br>• Cerebrovascular accident age <60<br>• Coronary artery disease age <60<br><br>MI, CVA, or coronary artery disease reported in a relative at an unknown age were not included.
Hereditary breast and ovarian cancer syndrome (*BRCA1, BRCA2*)	One or more of:<br>• Breast cancer (including DCIS)<br>• Ovarian cancer<br>• Prostate cancer<br>• Melanoma*<br>• Pancreatic cancer*<br><br>**BRCA2* only	One or more FDR, or multiple SDR history of:<br>• Breast cancer age <70^{+}<br>• Ovarian cancer^{+}<br>• Prostate cancer age <70<br>• Melanoma*<br>• Pancreatic cancer*<br><br>**BRCA2* only<br>$^{+}$Female SDRs through intervening males are treated as FDRs. Two FDRs at any age included. Breast cancer or prostate cancer reported in a relative at an unknown age, or any suspected cancers were not included.
Lynch syndrome (*MLH1*, *MSH2*, *MSH6*)	One or more of:<br>• Bowel cancer<br>• Bowel polyps<br>• Endometrial cancer<br>• Ovarian cancer<br>• Prostate cancer<br>• Ureter/kidney cancer<br>• Bladder cancer<br>• Brain tumour<br>• Sebaceous adenoma<br>• Pancreatic cancer	One or more FDR, or multiple SDR history of:<br>• Bowel cancer age <70<br>• Bowel polyps age <70<br>• Endometrial cancer<br>• Ovarian cancer<br>• Prostate cancer age <70<br>• Ureter/kidney cancer<br>• Bladder cancer<br>• Brain tumour<br>• Sebaceous adenoma<br>• Pancreatic cancer<br><br>Bowel or prostate cancer reported in a relative at an unknown age, or any suspected cancers, were not included. Three successive generations of bowel cancer at any age were included.

[1] Firth, Helen V., and Jane A. Hurst, *Oxford Desk Reference: Clinical Genetics and Genomics*, 2nd edition, Oxford Desk Reference Series (Oxford, 2017; online edn, Oxford Academic, 1 Oct. 2017)

<table>
<tr><td>Familial adenomatous polyposis, MYH-associated polyposis (APC, biallelic MUTYH)</td><td>One or more of:
Bowel cancer
Bowel polyps
Duodenal cancer
Duodenal polyps
Fundic gland polyps
Hepatoblastoma
Papillary thyroid cancer
Osteoma
Desmoid tumour
Sebaceous gland tumour/cyst
Congenital hypertrophy of the Retinal Pigment Epithelium (CHRPE)
Brain tumour
Adrenal gland adenoma
Dental anomalies</td><td>One or more FDR, or multiple SDR history of:
Bowel cancer age <70
Bowel polyps age <70
Duodenal cancer
Duodenal polyps
Fundic gland polyps
Hepatoblastoma
Papillary thyroid cancer
Osteoma
Desmoid tumour
Sebaceous gland tumour/cyst
Congenital hypertrophy of the Retinal Pigment Epithelium (CHRPE)
Brain tumour
Adrenal gland adenoma
Dental anomalies</td></tr>
<tr><td>Multiple endocrine neoplasia (MEN1, RET)</td><td>One or more of:
Parathyroid hyperplasia
Pituitary adenoma
Pancreatic adenoma
Insulinoma
Gastroma
VIPoma
Glucagonoma
Prostate cancer
Medullary thyroid cancer
Phaeochromocytoma
Adrenal cortical tumour
Lipoma
Mucosal neuroma
Marfanoid habitus
Medullated corneal nerve fibres
Diverticulae
Megacolon
Angiofibroma
Collagenoma</td><td>One or FDR or multiple SDR of:
Clinical diagnosis of MEN
Parathyroid hyperplasia
Pituitary adenoma
Pancreatic adenoma
Insulinoma
Gastroma
VIPoma
Glucagonoma
Prostate cancer
Medullary thyroid cancer
Phaeochromocytoma
Adrenal cortical tumour
Lipoma
Mucosal neuroma
Marfanoid habitus
Medullated corneal nerve fibres
Diverticulae
Megacolon
Angiofibroma
Collagenoma</td></tr>
<tr><td>von Hippel Lindau (VHL)</td><td>One or more of:
Retinal angioma
Cerebellar haemangioblastoma
Spinal cord haemangioblastoma
Renal cell carcinoma
Phaeochromocytoma
Renal cysts</td><td>One or more of:
Clinical diagnosis of VHL
Retinal angioma
Cerebellar haemangioblastoma
Spinal cord haemangioblastoma
Renal cell carcinoma
Phaeochromocytoma
Renal cysts</td></tr>
</table>

	• Pancreatic cysts<br>• Endolymphatic sac tumours<br>• Papillary cystadenoma of epididymis/broad ligament<br>• Pancreatic neuroendocrine tumour	• Pancreatic cysts<br>• Endolymphatic sac tumours<br>• Papillary cystadenoma of epididymis/broad ligament<br>• Pancreatic neuroendocrine tumour

Setting

100KGP[2] was a hybrid clinical research initiative, offering genome sequencing during routine clinical (NHS) care with mandatory clinical and genetic data capture and availability for future research and commercial use, and research governance. Participants were recruited either to the rare disease arm (affected individuals, including some with suspected genetic predisposition to cancer, and some healthy relatives in a trio strategy), or to the cancer arm offering paired tumour/germline sequencing in affected individuals. During recruitment, participants were offered a choice about a search for AFs. The AF gene list was available on the Genomics England website but not included in participant recruitment materials, which advised that the gene list was subject to change. Children were recruited using appropriate recruitment literature and offered screening for AFs in a smaller gene list; children, and heterozygosity for X-linked and recessive conditions are not included in the present study.

Health conditions associated with 100KGP AFs are cancer predisposition and FH, conditions which are underdiagnosed in clinical practice but for which evidence-based screening and/or interventions are available through the NHS. Pathogenic variants in the cancer-related AF genes predispose to cancer syndromes: *BRCA1/2* to breast, ovarian and prostate cancer, *MLH1*, *MSH2*, and *MSH6* to Lynch syndrome (adenomatous bowel polyps with potential for malignant transformation, bowel and gynaecological cancer); *APC* to FAP (bowel polyps and bowel cancer); biallelic *MUTYH* to MAP (bowel polyps and bowel cancer); *RET* and *MEN1* to MEN (tumours of the endocrine system); *VHL* to VHL (multisystem tumours). Pathogenic variants in the FH-related AF genes (*APOB*, *LDLR*, *PCSK9,* one variant in *APOE*) are associated with increased risk of premature atherothrombotic disease due to lifelong exposure to elevated low-density lipoprotein cholesterol (LDL-C). Recommended risk assessment and management for individuals with a heterozygous pathogenic variant in the NHS are listed in supplementary material.

The majority (91.8%) of 100KGP participants nationally answered 'yes' to the binary choice regarding AFs. Genomics England undertook bioinformatic analysis for variants predicted to result in loss of function, very rare

[2] https://www.genomicsengland.co.uk/initiatives/100000-genomes-project

missense variants, or classified as pathogenic or likely pathogenic in ClinVar[3]. Most putative AFs were released in five planned batches by Genomics England between August 2021 and March 2022; two further finalising batches were released after the study time frame. Putative AFs were released to NHS clinical laboratories for clinical-grade interpretation using ACMG criteria and confirmation; consensus-seeking communication between clinical laboratories was undertaken when interpretation bordered on likely pathogenic/uncertain significance. Only variants with pathogenic or likely pathogenic classification were retained. After removing variants already known to participants through standard-of-care or reported as 100KGP 'primary' findings, 100KGP participants in whom an AF was found were notified by NHS clinical services using either a standard letter which did not contain information about the specific AF, or a bespoke letter designed by clinical teams. When patients were deceased, attempts were made to contact relatives facilitated by participant preferred contacts listed on the 100KGP consent form.

Central and South Genomic Medicine Service Alliance (C&S GMS)[4] is one of seven alliances created by NHS England to embed genomics into routine care as part of the NHS Genomic Medicine Service. C&S GMS covers around one fifth the population of England and includes: West Midlands (Birmingham Women's and Children's NHS Foundation Trust, BWC, and University Hospitals Birmingham, UHB), Oxfordshire (Oxford University Hospitals NHS Foundation Trust, OUH) and Wessex (University Hospitals Southampton, UHS).

Cost calculations

Full details of all parameters and data sources for the cost calculations are provided in Table S3. Cost calculations were performed from a healthcare provider perspective, so included costs related to taking of consent, laboratory work, reporting of results by clinical scientists, genetic counsellor time pre-disclosure, administrative time to organise the disclosure process, and the costs of disclosing results in outpatient appointments in secondary care. The time horizon for the analysis was from the start of the initial consenting process up to and including the return of results in outpatient appointments in secondary care, with a cost year of 2022. Tests and medical interventions received after the disclosure of results were not included in this cost analysis. Cascade health service use beyond the index patient was also not included in this analysis. No cost conversions (e.g. between currencies) were necessary as part of this analysis.

For process 1, the time taken to consent patients for AFs and the type of staff who took consent were estimated based on the experience of clinical personnel at BWC and UHS. Costs were estimated separately for each centre,

---

[3] https://www.ncbi.nlm.nih.gov/clinvar/
[4] https://centralsouthgenomics.nhs.uk

taking into account the staff-mix at each centre, then a mean value was calculated and applied to all participants recruited in C&S GMS who elected for AFs.

For process 2, the cost of processing a genome through the Genomics England biopipeline to identify AFs was estimated at £56. This cost was applied to all participants recruited in C&S GMS who elected for AF. Bioinformatician time required alongside the pipeline costs was estimated for three categories of cases with AFs: those requiring no intervention, some intervention, and significant intervention.

For process 3, the staff time required to complete different tasks and the member of staff who would typically complete these tasks were estimated based on the process for managing AFs in OUH. Staff time was estimated separately for true AF cases, putative AF cases that were not reported, and cases with no findings. Consumable and equipment costs for this process were negligible, with two exceptions. First, the costs of sample transport were calculated based on actual practice in OUH. Second, we assumed that all true positive cases underwent validation using Sanger sequencing, at a cost of £130 per test. To calculate the total cost associated with process 3, the estimated laboratory cost for each type of case was multiplied by the total number of cases in this category in the C&S GMS.

For process 4, the staff time required to complete this task, and the members of staff who contributed to this task, were estimated based on historic practice at UHS and OUH. Costs were estimated separately for 'positive' and 'negative' AF letters, then multiplied by the number of letters sent out in each category. For 'positive' letters, tasks included reviewing the family file, generating the letter, booking the patient into a clinic, sending out the venue details and organising post-clinic letters. For 'negative' letters, system-wide tasks included finalising the letter content, training, periodic meetings to guide the process, set-up and liaison time with the supplier posting out the letters, genetic counsellor time to respond to queries from participants, and finally time to generate 'negative' letters for each participant.

Unit costs for processes 1-4 were extracted from published sources, including NHS Reference Costs[5]. Staff time was costed using NHS Agenda for Change salary scales published in April 2022[6]. Hourly rates were calculated under the assumption that staff worked 37.5 hours per week for 46 weeks of the year. All staff costs were inflated by 20% to account for national insurance and superannuation.

[5] National Schedule of NHS Costs - Year 2021/22 - NHS Trusts and NHS Foundation Trusts. Available from https://www.england.nhs.uk/costing-in-the-nhs/national-cost-collection/

[6] NHS Agenda for Change Pay Scales for 2022/23. Available from: https://www.nhsemployers.org/articles/pay-scales-202223

For process 5, the disclosure secondary care resource use data extracted from medical records for the study cohort were converted to match the format of episodes recorded in Hospital Episodes Statistics datasets (with an episode defined as a single outpatient appointment). For this analysis, we included both attended and not attended outpatient episodes, given the significant amount of pre-appointment work required in either scenario. Following data cleaning, resource use data were processed using the 2021-22 Reference Costs Grouper[7] to attach Healthcare Resource Group codes to each episode. These codes were then used to attach Reference Costs to each episode, using published NHS Reference Costs from 2021-22[8]. Mean episode costs were calculated both overall, and by type of appointment, type of AF, gender, NHS Trust and appointment age. Mean resource use per patient was also calculated (number of episodes per patient) as well as mean total costs per patient across all episodes. Mean values were also calculated for patient subgroups as above, and a total cost was calculated across all participants. As disclosure-related healthcare data were only available for a proportion of individuals with an AF, we scaled up the total cost by 1.76 (157/89) to estimate disclosure-related healthcare costs across the whole population receiving an AF.

Table S4 presents the results of the one-way sensitivity analysis. Most parameter variations had no effect on the study results. The one exception was the cost of the Genomics England Additional Finding pipeline: when this increased from £56 per genome to £84 per genome, the cost per participant with a putative AF increased from £3,615 to £4,892, and the cost per new AF identified increased from £8,680 to £11,746. When this cost reduced from £56 per genome to £28 per genome, the cost per participant with a putative AF decreased from £3,615 to £2,338, and the cost per new AF identified decreased from £8,680 to £5,613.

[7] HRG4+ 2021/22 National Costs Grouper. Available from: https://digital.nhs.uk/services/national-casemix-office/downloads-groupers-and-tools/grouper-and-tools-archive/reference-costs-groupers

[8] 2021/22 National Cost Collection Data Publication. Available from: https://www.england.nhs.uk/publication/2021-22-national-cost-collection-data-publication/

Table S3. Parameters and data sources used in the cost calculations

Costing process	Parameter	Base case value [a]	Values used in sensitivity analysis	Notes	Source
1. Consent	Band 5 staff member (e.g. Trained consenter) – cost per hour	£20.87	-		NHS Pay Scales 2022/23[6]
	Band 6 staff member (e.g. Genetic counsellor) – cost per hour	£25.84	-		NHS Pay Scales 2022/23
	Band 7 staff member (e.g. Research nurse) – cost per hour	£31.07	-		NHS Pay Scales 2022/23
	Consultant – cost per hour	£72.17	-		NHS Pay Scales 2022/23
	Time taken to consent	10 minutes	5 minutes, 15 minutes	Average time taken to consent in BWC and UHS	Local observations
2. Genomic analysis	Cost of the Genomics England Additional Finding pipeline	£56.00	£28, £84		Genomics England
	Band 7 staff member (e.g. Bioinformatician) – cost per hour	£31.07	-		NHS Pay Scales 2022/23
	Time taken to process cases requiring no intervention	5 minutes	2.5 minutes, 7.5 minutes	80% of cases	Genomics England
	Time taken to process cases requiring some intervention	30 minutes	20 minutes, 40 minutes	15% of cases	Genomics England
	Time taken to process cases requiring significant intervention	60 minutes	40 minutes, 80 minutes	5% of cases	Genomics England
3. Variant confirmation and interpretation	Band 2 staff member (e.g. Administrative staff) – cost per hour	£14.47	-		NHS Pay Scales 2022/23
	Band 6 or 7 staff member (e.g. Clinical scientist) – cost per hour	£28.31	-		NHS Pay Scales 2022/23
	Band 8 staff member (e.g. Senior clinical scientist) – cost per hour	£48.80	-		NHS Pay Scales 2022/23
	Band 8a staff member – cost per hour	£35.88	-		NHS Pay Scales 2022/23

Costing process	Parameter	Base case value [a]	Values used in sensitivity analysis	Notes	Source
	Sanger sequencing	£130	£100, £160		OUH
	Postage costs for sample transport, per package	£3.69	£1.85, £5.54	Average cost across the whole of the C&S GMSA	C&S GMSA
	Analysis and reporting time – true positive AFs	85 minutes	60 minutes, 110 minutes		OUH
	Analysis and reporting time – false positive AFs, variants of unknown significance, benign findings	75 minutes	50 minutes, 100 minutes		OUH
	Analysis and reporting time – previously reported variants	15 minutes	7.5 minutes, 22.5 minutes		OUH
	Analysis and reporting time – no findings	4 minutes	2 minutes, 6 minutes		OUH
4. Communication of results	Band 2 staff member (e.g. Administrative staff) – cost per hour	£14.47	-		NHS Pay Scales 2022/23
	Band 4 staff member – cost per hour	£17.47	-		NHS Pay Scales 2022/23
	Band 7 staff member (e.g. Research nurse) – cost per hour	£31.07	-		NHS Pay Scales 2022/23
	Band 8 staff member (e.g. Senior clinical scientist) – cost per hour	£48.80	-		NHS Pay Scales 2022/23
	Band 8a staff member – cost per hour	£35.88	-		NHS Pay Scales 2022/23
	Consultant – cost per hour	£72.17	-		NHS Pay Scales 2022/23
	Cost of generating a positive AF letter	£113.32	£56.66, £169.98		Calculated
	Cost of generating a negative AF letter	£0.27	£0.14, £0.41		Calculated
	Cost of sending a letter	£0.70	£0.35, £1.05		OUH
5. Disclosure consultations	Non-Admitted Face-to-Face Attendance, Follow-up, Clinical Genetics Service, Consultant-led	£433.14	-		NHS Reference Costs

Costing process	Parameter	Base case value [a]	Values used in sensitivity analysis	Notes	Source
	Non-Admitted Face-to-Face Attendance, Follow-up, Clinical Genetics Service, Non Consultant-led	£400.91	-		NHS Reference Costs
	Non-Admitted Face-to-Face Attendance, First, Diabetes Service, Consultant-led	£290.27	-		NHS Reference Costs
	Non-Admitted Face-to-Face Attendance, First, Clinical Genetics Service, Consultant-led	£694.36	-		NHS Reference Costs
	Non-Admitted Face-to-Face Attendance, First, Clinical Genetics Service, Non Consultant-led	£437.63	-		NHS Reference Costs
	Non-Admitted Face-to-Face Attendance, First, General Internal Medicine Service, Non Consultant-led	£108.57	-		NHS Reference Costs
	Non-Admitted Non Face-to-Face Attendance, Follow-up, Clinical Genetics Service, Non Consultant-led	£342.55	-		NHS Reference Costs
	Non-Admitted Non-Face-to-Face Attendance, First, Clinical Genetics Service, Consultant-led	£503.35	-		NHS Reference Costs
	Non-Admitted Non-Face-to-Face Attendance, First, Clinical Genetics Service, Non Consultant-led	£492.74	-		NHS Reference Costs
All processes	Multiplier for National Insurance and Superannuation	1.2	1, 1.4	All hourly costs for staff members multiplied by this amount to reflect National Insurance and Superannuation costs.	Assumption
	Working weeks per year	46	42, 50		Assumption
	Working hours per week	37.5	35, 40		Assumption

AF = additional finding. BWC = Birmingham Women's and Children's NHS Foundation Trust. C&S GMSA = Central and South Genomic Medicine Service Alliance. OUH = Oxford University Hospitals NHS Foundation Trust. UHB = University Hospitals Birmingham. UHS = University Hospitals Southampton [a] All staff costs have been adjusted to incorporate National Insurance and Superannuation costs. Costs per hour are calculated assuming 46 working weeks in a year, and 37.5 hour working weeks.

Table S4. One-way sensitivity analysis results

Parameter	Variation	Cost across the C&S GMS						Cost per participant with AF panel applied	Cost per participant with a putative AF	Cost per new AF identified
		Consent	Genomic analysis	Variant confirmation and interpretation	Communication of results	Disclosure consultations	Total cost			
Base case analysis	-	£85,981	£1,065,261	£79,773	£44,594	£87,078	£1,362,687	£79	£3,615	£8,680
Time taken to consent for AFs (10 minutes)	5 minutes	£42,990	£1,065,261	£79,773	£44,594	£87,078	£1,319,697	£77	£3,501	£8,406
	15 minutes	£128,971	£1,065,261	£79,773	£44,594	£87,078	£1,405,677	£82	£3,729	£8,953
Cost of Genomics England AFs pipeline (£56)	£28	£85,981	£583,829	£79,773	£44,594	£87,078	£881,255	£51	£2,338	£5,613
	£84	£85,981	£1,546,693	£79,773	£44,594	£87,078	£1,844,119	£107	£4,892	£11,746
Time taken to process cases requiring no intervention (5 minutes)	2.5 minutes	£85,981	£1,047,453	£79,773	£44,594	£87,078	£1,344,879	£78	£3,567	£8,566
	7.5 minutes	£85,981	£1,083,069	£79,773	£44,594	£87,078	£1,380,495	£80	£3,662	£8,793
Time taken to process cases requiring some intervention (30 minutes)	20 minutes	£85,981	£1,051,905	£79,773	£44,594	£87,078	£1,349,331	£78	£3,579	£8,594
	40 minutes	£85,981	£1,078,617	£79,773	£44,594	£87,078	£1,376,043	£80	£3,650	£8,765
Time taken to process cases requiring significant intervention (60 minutes)	40 minutes	£85,981	£1,056,357	£79,773	£44,594	£87,078	£1,353,783	£79	£3,591	£8,623
	80 minutes	£85,981	£1,074,165	£79,773	£44,594	£87,078	£1,371,591	£80	£3,638	£8,736

Parameter	Variation	Cost across the C&S GMS						Cost per participant with AF panel applied	Cost per participant with a putative AF	Cost per new AF identified
		Consent	Genomic analysis	Variant confirmation and interpretation	Communication of results	Disclosure consultations	Total cost			
Sanger sequencing cost (£130)	£100	£85,981	£1,065,261	£73,863	£44,594	£87,078	£1,356,777	£79	£3,599	£8,642
	£160	£85,981	£1,065,261	£85,683	£44,594	£87,078	£1,368,597	£80	£3,630	£8,717
Postage costs for sample transport, per sample (£3.69)	£1.85	£85,981	£1,065,261	£79,753	£44,594	£87,078	£1,362,667	£79	£3,615	£8,679
	£5.54	£85,981	£1,065,261	£79,794	£44,594	£87,078	£1,362,707	£79	£3,615	£8,680
Analysis and reporting time – true positive AFs (85 minutes)	60 minutes	£85,981	£1,065,261	£76,858	£44,594	£87,078	£1,359,772	£79	£3,607	£8,661
	110 minutes	£85,981	£1,065,261	£82,688	£44,594	£87,078	£1,365,602	£79	£3,622	£8,698
Analysis and reporting time – false positive AFs, variants of unknown significance, benign findings (75 minutes)	50 minutes	£85,981	£1,065,261	£78,691	£44,594	£87,078	£1,361,605	£79	£3,612	£8,673
	100 minutes	£85,981	£1,065,261	£80,856	£44,594	£87,078	£1,363,769	£79	£3,617	£8,686
Analysis and reporting time – previously reported variants (15 minutes)	7.5 minutes	£85,981	£1,065,261	£79,398	£44,594	£87,078	£1,362,312	£79	£3,614	£8,677
	22.5 minutes	£85,981	£1,065,261	£80,148	£44,594	£87,078	£1,363,062	£79	£3,616	£8,682
Analysis and reporting time – no findings (4 minutes)	2 minutes	£85,981	£1,065,261	£59,666	£44,594	£87,078	£1,342,579	£78	£3,561	£8,551
	6 minutes	£85,981	£1,065,261	£99,881	£44,594	£87,078	£1,382,795	£80	£3,668	£8,808
Cost of generating a positive AF letter (£113.32)	£56.66	£85,981	£1,065,261	£79,773	£35,699	£87,078	£1,353,791	£79	£3,591	£8,623
	£169.98	£85,981	£1,065,261	£79,773	£53,490	£87,078	£1,371,583	£80	£3,638	£8,736

Parameter	Variation	Cost across the C&S GMS						Cost per participant with AF panel applied	Cost per participant with a putative AF	Cost per new AF identified
		Consent	Genomic analysis	Variant confirmation and interpretation	Communication of results	Disclosure consultations	Total cost			
Cost of generating a negative AF letter (£0.27)	£0.14	£85,981	£1,065,261	£79,773	£42,271	£87,078	£1,360,364	£79	£3,608	£8,665
	£0.41	£85,981	£1,065,261	£79,773	£46,917	£87,078	£1,365,010	£79	£3,621	£8,694
Cost of sending a letter (£0.70)	£0.35	£85,981	£1,065,261	£79,773	£38,576	£87,078	£1,356,669	£79	£3,599	£8,641
	£1.05	£85,981	£1,065,261	£79,773	£50,612	£87,078	£1,368,705	£80	£3,631	£8,718
Multiplier for National Insurance and Superannuation (1.2)	1	£71,651	£1,048,195	£70,753	£39,168	£87,078	£1,316,844	£77	£3,493	£8,388
	1.4	£100,311	£1,082,327	£88,794	£50,021	£87,078	£1,408,530	£82	£3,736	£8,972
Working weeks per year (46)	42	£94,169	£1,075,013	£84,928	£47,695	£87,078	£1,388,883	£81	£3,684	£8,846
	50	£79,102	£1,057,069	£75,443	£41,990	£87,078	£1,340,682	£78	£3,556	£8,539
Working hours per week (37.5)	35	£92,122	£1,072,575	£83,639	£46,920	£87,078	£1,382,334	£80	£3,667	£8,805
	40	£80,607	£1,058,861	£76,391	£42,559	£87,078	£1,345,496	£78	£3,569	£8,570

AF = additional finding. C&S GMSA = Central and South Genomic Medicine Service Alliance.

UK risk assessment and management recommendations for individuals with a heterozygous pathogenic variant

BRCA1/2

(Female) Breast screening: annual breast MRI from 30 years and annual mammography from 40. Breast MRI may start from age 25 for women with individualised 10-year breast cancer risk of ≥8%. Offer referral to breast surgery team to discuss risk-reducing bilateral mastectomy +/- reconstruction. Individualised risk assessment recommended. Offer referral to gynaecology team to discuss risk-reducing bilateral salpingo-oophorectomy after family completion if appropriate and no earlier than 35-40 years.

(Male) Prostate screening: *BRCA2*: annual PSA checks from 40 years. *BRCA1*: no recommended screening but should speak with their GP about prostate health.

(Male and female) Pancreatic screening: not currently recommend outside of research study

Lynch Syndrome

Bowel screening: colonoscopy every 2 years from age 25 (*MLH1 and MSH2*) or 35 (*MSH6*) to age 75. Gastric screening: one-off *Helicobacter pylori* screen +/- treatment. Chemoprevention: all individuals should discuss the use of aspirin 150mg OD (under 70kg) or 300mg (over 70kg) with their GP from age 25-65.

(Female) Offer referral to gynaecology team to discuss total abdominal hysterectomy with bilateral salpingo-oophorectomy after family completion if appropriate, and no earlier than 35 years.

Familial adenomatous polyposis

APC: colonoscopy every 1-3 years from the ages of 12-14, and gastroscopy/duodenoscopy from age 25. Risk-reducing surgery options include total colectomy with ileorectal anastomosis, or proctocolectomy and ileal pouch anal anastomosis. Surgical decision based on size of polyps, presence of high-grade dysplasia in present polyps, and significant increases in polyp burden in screening intervals.

MYH-associated polyposis

MUTYH (biallelic): colonoscopy annually from age 18-20 years and gastroscopy/duodenoscopy from age 35.

Von Hippel-Lindau

Surveillance should start in childhood and include examination of: retina, CNS, inner ear, kidneys, neuroendocrine glands and pancreas. Usually managed by a specialised VHL clinic[9].

Multiple endocrine neoplasia

MEN1 (*MEN1*): Surveillance usually managed through specialist endocrinology clinic: genotype positive individuals are offered lifelong follow-up from childhood. This includes physical examination, biochemical tests and radiology screening for the three main mainfestations: parathyroid disease, duodeno, pancreatic, neuroendocrine tumours and pituitary adenomas[10]

MEN2 (*RET*): surveillance often managed through specialist endocrinology oncology MDT clinics, comprising annual review with biochemical testing and radiology imaging for medullary thyroid cancer (MTC) phaeochromocytoma and parathyroid disease. Thyroidectomy indicated from childhood or young adulthood (age for thyroidectomy guided by MTC risk level associated with specific variant and serum calcitonin levels)[11].

Familial hypercholesterolemia (heterozygous)

Lifelong lipid-lowering therapy aiming to achieve at least a 50% reduction in LDL cholesterol concentration from the baseline measurement; lifestyle advice including dietary and smoking avoidance. Annual review[12].

---

[9] Binderup et al. von Hippel-Lindau disease: Updated guideline for diagnosis and surveillance. European Journal of Medical Genetics (2022) Volume 65, Issue 8.

[10] MEN syndromes: https://bestpractice.bmj.com/topics/en-gb/866

[11] Wells et al. Revised American Thyroid Association Guidelines for the Management of Medullary Thyroid Carcinoma. Thyroid 2015 Jun;25(6):567-610.

[12] https://cks.nice.org.uk/topics/hypercholesterolaemia-familial/

Patient notification document – SAFE

What is this leaflet about?

Thank you for participating in the Genomics England 100,000 Genomes Project. You are receiving this leaflet because Genomics England has found an "additional finding" in your sample. We understand that it might be far from easy to receive this type of news.

It is the first time that the NHS has used genetic information in this way. We are a small team of researchers who are studying what happens next for people who receive an additional finding, and how the NHS handles this information. We have set up a study called SAFE (Secondary/Additional Findings Evaluation). With your help, the SAFE study can start to better understand this new area of medicine.

This leaflet explains more about the SAFE study, how you can discuss any concerns and opt out if you wish.

What are additional findings?

Additional findings are changes or glitches in a particular gene that may increase the chance of having certain health conditions. They are called "additional" as they are separate from a family's original reason for joining the 100,000 Genomes Project. Genomics England carefully selected a list of genes in which to look for additional findings. Glitches in these genes can either cause, or increase a person's likelihood of having, a particular health condition.

These glitches are "actionable" because actions can be taken if the person and their healthcare team know about them.

What is the study about?

The SAFE study will use information from the medical records of people who receive an additional finding, and interview some of them, to start to understand:

- the link between additional findings (genetics) and health conditions (signs and symptoms)
- how the NHS manages additional findings
- how additional findings impact upon patients

Understanding what happens after people are told about additional findings will contribute to national discussions on how best to use this type of genetic findings to deliver patient-centred care in the future. By doing so, we can help ensure that other families receiving this type of news in the future are receiving the highest quality care based on evidence.

Information from your medical record, relating to the additional finding, would be incredibly helpful to answer some of these questions. For example, any appointments or tests that your care team might arrange in the next twelve months (the length of the study). To collect this information from people who receive an additional finding, we would like to access your medical records at the hospital.

Who is the study team, and how will you look after my information?
We are a small team of experienced researchers. Some of our team also have clinical experience working in the NHS, talking to patients and their families about genetics in medicine. The SAFE study has Research Ethics Committee approval.

Information security is paramount to us. We will not store or copy any information that could be used to identify you. The three researchers who will access your medical records have been vetted by the NHS, and they will do so only to ensure they relate to the right person; the researchers will store only de-identified information on secure NHS servers.

A Privacy Notice can be found at rdm.ox.ac.uk/safe-study

Why do you need to ask about this? Isn't it covered by the 100,000 Genomes Project consent?
As part of your participation in the 100,000 Genomes Project, Genomics England can collect some information from your medical record and put it in a patient data library (sometimes called the "research environment"). This information does not identify people individually. However, the information needed for this study may not all be collected in the patient data library, and it may take a significant amount of time to be collected and available. For these reasons, we would like to access your medical records to ensure we are collecting the right medical information and doing so in a time frame that allows this research to be used to contribute to improving future patient care.

What do I need to do now?
If you are happy for us to look at your medical record relating only to your additional finding, and to analyse that information together with the information of other people who receive an additional finding, you do not need to do anything.

However, if you do not wish for us to access the information in your medical record for this research, you can opt out at any time. If you opt out of your information being used for the SAFE study, this will not impact the clinical care you receive, and you would stay in the 100,000 Genomes Project. To opt out of your information being accessed for SAFE, please visit this website rdm.ox.ac.uk/safe-study– and fill in this form; you'll be asked to input your NHS number (this is a 10-digit number which can be found on hospital letters) and tick the opt-out box.

Alternatively, you can opt out by calling 01865 226017. We will only ask you to provide your NHS number. You do not have to provide a reason for your decision to opt out.

If you have any questions, we will be happy to discuss further on 01865 226017.

Thanks to patients and families like you, knowledge of genetic medicine is improving at a rapid pace. We are very grateful to all participants for their invaluable role in our work. Together, we can contribute to our ever-expanding knowledge of the interplay between genetics and health and how the NHS can best support individuals receiving this type of genetic information.
